# Deaths “due to” COVID-19 and deaths “with” COVID-19 during the Omicron variant surge, among hospitalized patients in seven tertiary-care hospitals, Athens, Greece

**DOI:** 10.1038/s41598-025-98834-y

**Published:** 2025-04-21

**Authors:** Dimitrios Basoulis, Krystalenia Logioti, Ioanna Papaodyssea, Marios Chatzopoulos, Panagiota Alexopoulou, Panagiotis Mavroudis, Vassiliki Rapti, Vassiliki Poulia, Stamatina Samara, Vassiliki E. Georgakopoulou, Maria N. Gamaletsou, Christos Michailidis, Garyfallia Poulakou, Theano Kontopoulou, Vasileios Papastamopoulos, Georgios Chrysos, Maria Chini, Anastasia Antoniadou, Nikolaos V. Sipsas

**Affiliations:** 1 Infectious Diseases / COVID-19 Unit, General Hospital of Athens Laiko, Athens, Greece; 2 “Korgialeneio-Benakeio” Red Cross General Hospital, Athens, Greece; 3 Evaggelismos General Hospital, Athens, Greece; 4 G. Gennimatas General Hospital, Athens, Greece; 5 Attikon General Hospital, Athens, Greece; 6 Tzaneio General Hospital, Piraeus, Greece; 7 Sotiria Chest Disease Hospital, Athens, Greece; 8 Medical School, National and Kapodistrian University of Athens, Athens, Greece

**Keywords:** COVID-19, Hospital mortality, Cause of death, Death certificates, Greece, Infectious diseases, Viral infection, Epidemiology

## Abstract

In Greek hospitals, all deaths with a positive SARS-CoV-2 test are counted as COVID-19 deaths. Our aim was to investigate whether COVID-19 was the primary cause of death, a contributing cause of death or not-related to death amongst patients who died in hospitals during the Omicron surge and were registered as COVID-19 deaths. Additionally, we aimed to analyze the factors associated with the classification of these deaths. We retrospectively re-viewed all in-hospital deaths, that were reported as COVID-19 deaths, in 7 hospitals, serving Athens, Greece, from January 1, 2022, until August 31, 2022. We retrieved clinical and laboratory data from patient records. Each death reported as COVID-19 death was characterized as: (A) death “due to” COVID-19, or (B) death “with” COVID-19. We reviewed 530 in-hospital deaths, classified as COVID-19 deaths (52.4% males; mean age 81.7 ± 11.1 years). We categorized 290 (54.7%) deaths as attributable or related to COVID-19 and in 240 (45.3%) deaths unrelated to COVID-19 In multivariable analysis The two groups differed significantly in age (83.6 ± 9.8 vs. 79.9 ± 11.8, *p* = 0.016), immunosuppression history (11% vs. 18.8%, *p* = 0.027), history of liver disease (1.4% vs. 8.4%, *p* = 0.047) and the presence of COVID-19 symptoms (*p* < 0.001). Hospital stay was greater in persons dying from non-COVID-19 related causes. Among 530 in-hospital deaths, registered as COVID-19 deaths, in seven hospitals in Athens during the Omicron wave, 240 (45.28%) were reassessed as not directly attributable to COVID-19. Accuracy in defining the cause of death during the COVID-19 pandemic is of paramount importance for surveillance and intervention purposes.

**Supplementary Information:**

The online version contains supplementary material available at 10.1038/s41598-025-98834-y.

## Introduction

Death is the most reliable hard outcome used to assess the impact of a pandemic on public health and monitor its evolution. Researchers have mentioned an information bias related to case and mortality definitions for COVID-19, that can vary among studies and sometimes within studies^1^. Therefore, accuracy in defining the cause of death during a pandemic, is of paramount importance for surveillance and intervention purposes. Since the beginning of the SARS-CoV-2 pandemic, there has been an ongoing debate on the definition of the COVID-19-attributable death. The European Center for Disease Control (ECDC) uses the guidelines issued by the World Health Organization (WHO), defining for surveillance purposes as COVID-19-attributable deaths all deaths “resulting from a clinically compatible illness in a probable or confirmed COVID-19 case, unless there is a clear alternative cause of death that cannot be related to COVID disease (e.g., trauma)”^2^. In the UK, Denmark, and other countries all deaths, for which a positive SARS-CoV-2 PCR test was recorded within the 30 days prior to the date of death, were registered as COVID-19 deaths^3–5^.

In Greece, a more concise and simple definition was used, defining as COVID-19-associated death, any death occurring in a person with positive testing for SARS-CoV-2 at the time of death. Nevertheless, throughout the pandemic, none of the above definitions has been able to determine accurately who has died ‘from’ or ‘with’ COVID-19. All the above definitions served the surveillance purposes well, at least during the initial pandemic waves, including the Delta variant. Researchers from the UK reported that during the first pandemic waves, COVID-19 deaths were accurately attributed to the virus itself approximating an impressive 92-97% accuracy^5^. In Denmark, during the Delta wave, only an estimated 10-20% of deaths registered as COVID-19 deaths were in fact due to other, non-COVID-19-related, causes^4^.

However, in Greece and worldwide, the epidemiological characteristics of the pan-demic changed dramatically with the advent of the Omicron variant, in January 2022^6^, when a sharp increase in the number of cases, and a lesser increase in admissions and deaths were recorded^7^. The higher infectivity^8,9^ and lower morbidity of the new variant, associated with lower risks of COVID-19–related hospitalization and death^10–12^, along with the high percentage of vaccination coverage of the population achieved at the time in Greece^13^ made plausible that a substantial percentage of hospitalized patients with comorbidities, who died with a positive test for SARS-CoV-2 and were registered as COVID-19 deaths, in fact died from other causes “with” COVID-19, and not “be-cause of” COVID-19.

Researchers have attempted to dissect the COVID-19 mortality and differentiate deaths “due to” COVID-19, where SARS-CoV-2 infection was the direct cause of death or triggered a series of events that ultimately led to death, from deaths “with” COVID-19, when the SARS-CoV-2 infection has nothing to do with the fatal outcome. These studies were based on data from death certificates, a method with limitations and potential inaccuracies^14,15^.

The aim of this study was to assess whether in-hospital deaths, registered as COVID-19-associated deaths, in seven tertiary-care hospitals in the greater area of Athens, Greece, during the Omicron surge, were attributed to COVID-19 or to other causes. Additionally, we aimed to analyze the factors associated with the classification of these deaths. To avoid the shortcomings of death certificates, we also examined the chart file of each patient and interviewed the caring physicians.

## Methods

### Study design

This is a retrospective observational study. Deaths registered as COVID-19-related were identified from the death certificates records of participating hospitals. In Greece, a paper copy of all death certificates issued by a hospital is kept in the hospital archives. As a parallel database, researchers used the Hellenic National Archive of COVID-19, a digital archive where physicians enter epidemiological, and clinical information, as well as out-comes for patients treated in hospitals for COVID-19. All patients, who died in the participating hospitals between 1st January 2022 and 31st August 2022, with COVID-19 being mentioned on their death certificate, and consequently registered as COVID-19 death, were included in the study. This study has received ethical approval from the Ethical Review Boards of participating hospitals. Informed consent was waived as the data was anonymized when included in the database for analysis and no risk or harm would come to participants. All methods used for this study were performed in accordance with institutional and national guidelines and in accordance with the Declaration of Helsinki regarding medical research. This article is a revised and expanded version of a paper entitled “Deaths of and with COVID-19 during the Omicron surge in seven tertiary care hospitals, Athens, Greece”, which was presented at the 33rd European Congress of Clinical Microbiology and Infectious Diseases, Copenhagen, Denmark^16^.

### Classification of recorded deaths

We categorized all study deaths into two groups: (a) deaths “due to” COVID-19, where the infection was either the direct or sole cause of death, or it triggered a sequence of events that ultimately led to death, and (b) deaths “with” COVID-19, where the death was unrelated to the infection. To classify each death, we used data from three sources: (a) the death certificate, (b) the chart file of the patient (paper and electronic files) and (c) we interviewed the caring physician with a structured questionnaire. We extracted epidemiological, clinical, and treatment data, including demographics, comorbidities, vaccination status, the reason for admission to the hospital, in-hospital transmission of SARS-CoV-2, the department where the patient was admitted, clinical signs and symptoms of COVID-19, laboratory and imaging findings attributed to COVID-19, need for supplementary oxygen, COVID-19 specific treatment (including early antiviral treatment for high-risk patients), and outcomes. We ascertained the presence of the following comorbidities: chronic obstructive pulmonary disease, diabetes, asthma, chronic kidney disease, liver disease or cirrhosis, autoimmune disorder, immunocompromised status (e.g., HIV infection, solid organ transplantation), rheumatoid arthritis, inflammatory bowel disease, ischemic heart disease, congestive heart failure, and transient ischemic attack or stroke. We also determined each person’s modified Charlson Comorbidity Index score. Then, the senior physician, who was the caring physician for the specific patient before death, reviewed the data and by answering to a structured questionnaire provided his/her opinion on the cause of death. Finally, two senior researchers, each with experience treating over 2,500 COVID-19 patients, served as independent reviewers. They carefully considered all available data, including the opinion of the attending physician and their own medical judgment, to classify each death into one of three categories, as outlined above. More specifically, we characterized a death as “due to” COVID-19 if the patient had signs, symptoms and laboratory findings of COVID-19 at the time of death, including pneumonia, confirmed by imaging findings, need for supplemental oxygen, receiving COVID-19 specific treatment, and had no other clear cause of death. For example, the in-hospital death of a patient admitted with recent-onset fever, hypoxia, a positive SARS-CoV-2 test, diffuse chest X-ray infiltrates, and a need for supplemental oxygen, who was treated with standard COVID-19 protocols but died despite treatment, was classified as a death “due to” COVID-19. As death “with” COVID-19 we characterized deaths of patients who were admitted for another reason, with no signs and symptoms attributed to COVID-19, no need for COVID-19 specific treatment, with the exception of early antiviral treatment to prevent progression in high-risk patients and had another obvious cause of death. As an example, a patient admitted for routine surgery, who tested positive for SARS-CoV-2 without showing any COVID-19 signs or symptoms, received no COVID-19 treatment, and died from a confirmed surgical complication, was classified as a death “with” COVID-19. Finally, COVID-19 was characterized as “contributing to” death, in patients with another clear cause of death but simultaneously with signs, symptoms and laboratory findings of COVID-19. This category has been incorporated into the deaths “due to” COVID-19 category, in the analysis.

### Statistical analysis

Descriptive statistics are presented as counts (%) for categorical variables and as medians [25th, 75th percentile] for non-normally distributed continuous variables or as means ± standard deviation (SD) for normally distributed continuous variables. Normality of distribution was examined using the Kolmogorov–Smirnov test. Group comparisons were performed using Student’s t-test and Mann Whitney U test for normally and non-normally distributed variables respectively, chi-square for categorical variables.

Multivariable analyses were performed using binary logistic regression. All variables with a p-value < 0.1 in the univariate analysis, as well as variables that would be reasonable to be added in the model based on known literature, were included^17^. We did not apply multiple comparison corrections for the logistic regression model. Each variable included was selected based on biological plausibility and prior evidence of potential association with COVID-19 mortality classification. Results of the logistic regression are presented as Odds Ratios (OR), with 95% confidence intervals (CI). Statistical findings with a p-value < 0.05 were considered statistically significant. The analysis was performed using SPSS Statistics for Windows, Version 25.0 (2017, Armonk, NY, IBM Corp.).

## Results

We reviewed a total of 530 in-hospital deaths, registered as COVID-19 deaths, over the study period. Demographic and clinical data are shown on Table 1. Of note, in our population, only 12 (2.3%) patients reported a previous episode of SARS-CoV-2 infection. The vast majority (95.6%) of our patient population had at least one comorbidity. After reviewing the death certificates, medical charts, and interviewing the attending physicians, we concluded that in 240 (45.3%) of the 530 reviewed cases, COVID-19 was not related to the death (deaths “with” COVID-19). In 133 (25.1%) cases, COVID-19 was determined to be the direct cause of death, while in 157 (29.6%) cases, COVID-19 was not the primary cause but contributed to the chain of events leading to death. These two latter categories combined accounted for 290 (54.7%) deaths classified as “due to” COVID-19.

**Table 1:** Basic demographic and clinical information of the study population.

	*N* = 530
Age	81.93 ± 10.9
Female gender	260 (49.1)
Medical History	
CAD/CHF	238 (44.9)
COPD/asthma	66 (12.5)
Solid organ neoplasm	98 (18.5)
Hematological cancer	38 (7.2)
Autoimmune disease	26 (4.9)
Diabetes	138 (26)
Renal disease	77 (14.5)
Liver disease	24 (4.5)
Neurological disease	228 (43)
Fracture	40 (7.5)
Immunosuppression	77 (14.5)
Transplant recipient	5 (0.9)
Atrial fibrillation	83 (15.7)
Age-adjusted Charlson score	6 [5–7]
Vaccination status	
Unvaccinated/partially vaccinated	220 (41.5)
Fully vaccinated	107 (20.2)
Boosted (> 2 doses)	198 (37.4)
Diagnosis	
Hospital transmission	108 (20.4)
Days from diagnosis to admission	0 [0–4]
Admission department	
Infectious Diseases	326 (61.5)
Internal Medicine	167 (31.5)
Other	37 (7)
Clinical information	
Signs/symptoms	444 (83.8)
Cough	40 (7.5)
Elevated ferritin	52 (9.8)
Lymphopenia	181 (34.2)
Hypoxia	180 (34)
Shortness of breath	140 (26.4)
Fever	197 (37.2)
Received treatment	411 (77.5)
Oxygen support	
No oxygen	97 (18.3)
Nasal cannula	49 (9.2)
Mask	334 (63)
HFNC/NIV	19 (3.6)
Mechanical ventilation	26 (4.9)
Remdesivir treatment	
No treatment	237 (44.7)
3-day prophylaxis	26 (4.9)
5-day treatment	262 (49.4)
Dexamethasone	332 (62.6)
Immunomodulators	
None	495 (93.4)
Tocilizumab	18 (3.4)
Anakinra	6 (1.1)
Baricitinib	5 (0.9)
Nirmatrelvir/ritonavir	3 (0.6)
Outcomes	
Days from admission to death	8 [4–15]
Death attributed or related to COVID-19	290 (54.7)
Death not related to COVID-19	240 (45.3)

Data is presented as N (%) or mean ± SD for continuous normally distributed variables or median [interquartile range 25th–75th] for continuous non-normally distributed variables. CAD = Coronary artery disease, CHF = congestive heart failure, COPD = chronic obstructive pulmonary disease, HFNC = high flow nasal cannula, NIV = non-invasive ventilation.

In Greece, the death certificate contains two distinct categories of death causes: condi-tions that directly caused the death and conditions that contributed to death. COVID-19 has been listed as direct cause of death in 204 of the study certificates and as contributing factor in 324 death certificates. In both cases, deaths were registered officially as COVID-19 deaths. Among the 204 deaths where COVID-19 was listed as direct cause of death on the death certificate, after our evaluation only 132 (64.7%) deaths were attributed to COVID-19; in 71 deaths (34.8%) COVID-19 was not the direct cause but contributed to death, and in only one case we classified death as not related to COVID-19. On the other hand, among the 324 deaths where COVID-19 was listed on the death certificate as contributing factor, after our evaluation, one death was attributed to COVID-19, in 85 (26.2%) cases COVID-19 was not the direct cause but a contributing factor and in 239 (73.5) cases we considered COVID-19 as not related to death. Among the 240 patients dying “with” COVID, leading cause of death was bacterial sepsis/septic shock (105/240), followed by aspiration pneu-monia (63/240), acute renal failure (10/240), stroke (15/240), heart failure (19/240), and solid organ or hematological cancers (28/240). We also investigated potential variation in classification discrepancies across participating hospitals, but the differences did not reach statistical significance, although different study hospitals serve different study populations.

We then compared patients whose death was attributed or related to COVID-19 (death “due to” COVID-19) vs. patients whose deaths were classified as not related to COVID-19 (deaths “with” COVID-19), to identify risk factors for death “with” COVID-19. In univariable analysis (Table 2), patients who died “with” COVID-19, compared to those who died “from” COVID-19 were significantly more likely to be younger (mean ± SD 79.9 ± 11.8 vs. 83.6 ± 9.8, *P* < 0.001), to have solid organ malignancy, end-stage liver disease, to be immunosuppressed and to be infected via hospital transmission. Conversely, patients who died “due to” COVID-19, were more likely to be older, to be admitted to an infectious disease ward, to have symptoms and laboratory compatible with COVID-19, to receive supplementary oxygen, and/or COVID-19-specific treatment.

**Table 2:** Comparison of demographic and clinical data between patients with deaths classified as “due to” and “with” COVID-19.

	Due to = 290	With = 240	*p*
Age	83.6 ± 9.8	79.9 ± 11.8	< 0.001
Female gender	150 (51.7)	110 (45.8)	0.191
Medical History			
CAD/CHF	141 (48.6)	97 (40.6)	0.066
COPD/asthma	41 (14.1)	25 (10.5)	0.235
Solid organ neoplasm	41 (14.1)	57 (23.8)	0.005
Hematological cancer	21 (7.2)	17 (7.1)	1
Autoimmune disease	17 (5.9)	9 (3.8)	0.315
Diabetes	67 (23.1)	71 (29.7)	0.091
Renal disease	40 (13.8)	37 (15.5)	0.621
Liver disease	4 (1.4)	20 (8.4)	< 0.001
Neurological disease	124 (42.8)	104 (43.5)	0.930
Fracture	18 (6.2)	22 (9.2)	0.247
Immunosuppression	32 (11)	45 (18.8)	0.013
Transplant recipient	2 (0.7)	3 (1.3)	0.662
Atrial fibrillation	49 (16.9)	34 (14.2)	0.471
Age-adjusted Charlson score	6 [5–7]	6 [5–8]	0.004
Vaccination status			0.086
Unvaccinated/partially vaccinated	133 (46.2)	87 (36.7)	
Fully vaccinated	53 (18.4)	54 (22.8)	
Boosted (> 2 doses)	102 (35.4)	96 (40.5)	
Diagnosis			
Hospital transmission	36 (12.5)	72 (30)	< 0.001
Days from diagnosis to admission	0 [0–4]	0 [0–5]	0.522
Admission department			< 0.001
Infectious Diseases	202 (69.7)	124 (51.7)	
Internal Medicine	80 (27.6)	87 (36.3)	
Other	8 (2.8)	29 (12.1)	
Clinical information			
Signs/symptoms	272 (94.4)	172 (72)	< 0.001
Cough	28 (9.7)	12 (5)	0.048
Elevated ferritin	21 (72.)	31 (12.9)	0.039
Lymphopenia	121 (41.7)	60 (25)	< 0.001
Hypoxia	128 (44.1)	52 (21.7)	< 0.001
Shortness of breath	91 (31.4)	49 (20.4)	0.005
Fever	114 (39.3)	83 (34.6)	0.279
Received treatment	284 (98.3)	170 (72)	< 0.001
Oxygen support			< 0.001
No oxygen	19 (6.6)	78 (33.1)	
Nasal cannula	30 (10.4)	19 (8.1)	
Mask	205 (70.9)	129 (54.7)	
HFNC/NIV	15 (5.2)	4 (1.7)	
Mechanical ventilation	20 (6.9)	6 (2.5)	
Remdesivir treatment			< 0.001
No treatment	99 (34.3)	138 (58.5)	
3-day prophylaxis	11 (3.8)	15 (6.4)	
5-day treatment	179 (61.9)	83 (35.2)	
Dexamethasone	236 (81.7)	96 (40.7)	< 0.001
Immunomodulators			0.003
None	263 (91)	232 (98.3)	
Tocilizumab	16 (5.5)	2 (0.8)	
Anakinra	5 (1.7)	1 (0.4)	
Baricitinib	5 (1.7)	0 (0)	
Nirmatrelvir/ritonavir	2 (0.7)	1 (0.4)	1
Days from admission to death	7 [4–13]	10 [3–18]	0.036

Data is presented as N (%) or mean ± SD for continuous normally distributed variables or median [interquartile range 25th–75th ] for continuous non-normally distributed variables. CAD = Coronary artery disease, CHF = congestive heart failure, COPD = chronic obstructive pulmonary disease, HFNC = high flow nasal cannula, NIV = non-invasive ventilation.

In multivariate analysis, by using binary logistic regression multivariable model, transmission of infection in the hospital, end-stage liver disease, immunosuppression and younger age were predictors that the ensuing death was “with” COVID-19 (Table 3).

**Table 3:** Multivariable binary logistic regression model to predict death “with” COVID-19.

	OR	95%CI	*p*
Age	0.98	0.95–0.99	0.016
Female gender	0.99	0.67–1.46	0.965
Solid organ cancer	0.89	0.44–1.81	0.746
Liver disease	3.32	1.02–10.85	0.047
Age adjusted Charlson	1.23	0.69–2.18	0.490
Immunosuppression	1.19	1.02–1.38	0.027
Vaccination status			
Unvaccinated/partially	ref	ref	ref
Fully	1.17	0.7–1.95	0.553
Boosted	1.03	0.67–1.58	0.906
Hospital transmission	2.3	1.39–3.81	0.001
Signs and symptoms	0.2	0.11–0.37	< 0.001

OR: Odds Ratio, CI: Confidence Interval, ref.: reference value.

## Discussion

In this retrospective, observational study, we found that of 530 in-hospital deaths, who have been registered as COVID-19 deaths, in seven Greek hospitals during the Omicron wave, only 290 (54.7%) were “due” to COVID-19. The rest were found to be deaths “with” COVID-19. This is the first study based not solely on death certificates, but also on data extracted by expert physicians from the chart file of each individual patient, and on the opinion of the caring physician. We found that patients who died “with” COVID-19 were more likely to be younger, to be admitted for any other cause but COVID-19, to have had malignancy or immunosuppression, and to have been infected in the hospital. Patients who did not exhibit COVID-19-related symptoms essentially did not experience the active effects of the infection. As a result, their deaths were not considered to be directly caused by COVID-19. This suggests that the absence of symptoms may indicate a lack of significant COVID-19-related impact, even if the virus was present.

Our findings suggest that the higher number of deaths reported form Greece during the Omicron wave was reflecting the higher level of transmission of the virus in the community and not necessarily higher morbidity or attributable mortality. Similarly, because community transmission was high, a person who was admitted for a reason unrelated to COVID-19 was more likely to have had a positive antigenic test for SARS-CoV-2 on admission, or to have been infected via in-hospital transmission and the ensuing death caused by the underlying disease to be misclassified as COVID-19 death. Assessing the importance of a positive antigenic or even PCR test on admission is not as simple as it might seem. The differences in prognosis in patients being admitted with an incidental positive test as opposed to true infection have been discussed in a study from the United States, where patients admitted for true infection were more likely to receive mechanical ventilation and die, but also the CDC classification system used on admission had a sensitivity of 83% in detecting true infections^18^. Onder et al., in Italy also discussed in an opinion paper that using COVID-19 positivity as the sole criterion for determining causality in relation to COVID-19 would only lead to an over-estimation of COVID-19 deaths^19^.

Deaths of older patients, admitted to dedicated COVID-19 Units, with signs and symptoms of active COVID-19, and who received COVID-19 specific treatment were more likely to be characterized as deaths “due” to COVID-19. On the contrary, patients who died “with” COVID-19 were younger but with more severe underlying diseases, were ad-mitted to non-COVID-19 wards, and were more likely to get infected after their admission in the hospital. Obviously, as the population had experienced a rise in cases during the Omicron era, many of which were less serious than previously, a rise was also seen in the proportion of deaths that were incorrectly recorded as COVID-19 deaths rather than deaths “with” COVID-19. Therefore, the daily figures describing COVID-19 deaths might have been less accurate than during the previous pandemic waves. In our instance, not-related deaths included deaths due to other infections, cardiovascular events (stroke and heart failure), aspiration pneumonia and solid organ or hematological cancers.

We report that 240 (45.28%) of the 530 deaths registered as GOVID-19 deaths were not related to COVID-19. Several reports from national cohorts have addressed disparities regarding attributed mortality and reporting of deaths during the pandemic. A review of COVID-19 deaths in Denmark, based on death certificates registered in the Danish Causes of Death Registry showed a similar proportion of deaths “with” COVID-19, not “due to” COVID-19, since the beginning of 2022. More specifically, probability calculations based on the weekly number of deaths and the incidence of COVID-19 community transmission, showed that, by the end of week 6 in 2022, almost 40% of all deaths occurring within 30 days after a COVID-19-positive PCR test should be classified as deaths “with” COVID-19 not “due to” COVID-19. Before the emergence of the Omicron variant, this proportion ranged from 10 to 20%. As a result, the 30-day COVID-19 death count increasingly over-estimated the fatalities “due to” COVID-19. In reality, the actual number of deaths directly caused by COVID-19 has risen only slightly, even as community transmission of the Omicron variant leads to a surge in cases^4^. In another study from Sweden, researchers examined the concordance between COVID-19 mortality statistics derived from clinical audit and death certificates in Ostergotland county. They assessed roughly 1000 deaths recorded as attributable or related to COVID-19 infection and found that in 24% of cases, were not related to COVID-19 infection at all^20^. Another example of bias in COVID-19 death reporting is found in China. Starting in December 2022, the definition for COVID-19 deaths was restricted to those specifically linked to respiratory disease. This change resulted in significantly lower reported death numbers compared to the country’s earlier estimates^21^.

A review by JPA Ioannidis highlighted that countries in Africa were likely to underreport deaths attributed to COVID-19, whereas in countries with intensive testing and heightened awareness, deaths were more likely to be reported as COVID-19-related early in the pandemic. The review also noted that while excess death estimates provide a more reliable metric, they could still be influenced by indirect effects of the pandemic, such as missed cancer treatments^22^. Nevertheless, comparing reported deaths with excess mortality data can offer valuable insights into the quality of global mortality data and reveal differences between countries^23^. In a 2021 report, the authors commended France and Belgium for the quality of their mortality data and contrasted this with Tajikistan, where there was a significant undercount. Of particular interest was the case of Peru, where a change in the definition of cause of death regarding COVID-19 led to a dramatic improvement in data quality^23^. Our study has some limitations, including the absence of data on patients transferred to ICUs. Due to a relative shortage of ICU beds, younger patients with COVID-19-related complications were more likely to receive mechanical ventilation than older patients with multiple comorbidities. Consequently, not including ICU deaths may result in an under-estimation of fatalities “due to” COVID-19 rather than those “with” COVID-19. Furthermore, we did not include deaths that occurred after hospital discharge, which again could skew the results. As with the ICU deaths, not being able to record these deaths due to lack of access to death certificates and data pertaining to these deaths, could have affected our results. Another limitation is the lack of a control group, from the pre-Omicron era, as well as possible subjectivity in the assessment of cases. In our study, we included data from 7 major hospitals in Athens, Greece, reflecting accurately both the overall population of Athens but also of Greece as a whole. However, applicability to other countries should be avoided since both the healthcare system capacity and the intensity of pandemic waves differ between countries. Finally, our study covers several months at the onset of the Omicron wave. Given the emergence of newer variants, it is possible that our findings may not fully apply to subsequent strains.

On the other hand, our study was not based solely on death certificates but also on data extracted from chart files and the opinion of caring physicians, and the independent assessment of two experienced reviewers, making our classification of each death more accurate. The question of death ‘with’ or ‘due to” COVID-19 remains a central issue to understand the impact of the pandemic. This question cannot be answered with any certainty through the sole use of death certificates, particularly given their inherent limitations^14^. In many hospitals, junior doctors and not senior physicians can often be tasked with signing medical certificates of cause of death, without the availability of autopsies^15^. The different populations served in each hospital could also have determined the practices of physicians signing death certificates. For example, one of the participating hospitals has a liver, kidney and stem cell transplant units but neither cardiothoracic surgery not neurosurgery units, which might make physicians more prone to recognise infectious complications of immunosuppressants and less likely to attribute causes of death to surgical complications. Prioritizing the condition leading to death can prove difficult: it is affected by the experience of the clinician, their prior knowledge of the patient and the presence of several comorbidities that may compete or co-exist^15^. This reliance on physician judgement ultimately introduces potential subjectivity and differences of opinion between physicians, inter-rater variability. Performing autopsies for all deceased would have helped address this problem, but it would be unfeasible.

## Conclusions

In conclusion, we found that 45,28% of the deaths registered as COVID-19 deaths, in seven hospitals in Athens Greece, were reassessed as not directly attributable to COVID-19 in our analysis, but reflected the wide transmission of the Omicron variant in the community. However, to make reliable inferences about mortality from COVID-19, we must eliminate important biases that may lead to inaccurate conclusions, based on the use of inaccurate definitions.

## Electronic supplementary material

Below is the link to the electronic supplementary material.

Supplementary Material 1

## Data Availability

Data is available at the Pergamos Repository affiliated with the National and Kapodistrian university of Athens. https://pergamos.lib.uoa.gr/uoa/dl/frontend/el/home/research/item/3429316.
