# Supplementary material for: Deaths “due to” COVID-19 and deaths “with” COVID-19 during the Omicron variant surge, among hospitalized patients in seven tertiary-care hospitals, Athens, Greece
Source: Sci Rep. 2025 Apr 21;15:13728. doi: 10.1038/s41598-025-98834-y (PMC12012217; doi:10.1038/s41598-025-98834-y)
Supplement: Supplementary file 1 — Supplementary Material 1 [file 41598_2025_98834_MOESM1_ESM.docx]

**Research Protocol (v.2)**

**Study on Mortality Observed in Patients Hospitalized in Regular Wards with SARS-CoV-2 Omicron Variant Infection in Greece:** **Deaths DUE TO COVID-19 and Deaths WITH COVID-19**

**Compiled by:** Nikolaos Sypsas, Professor, National and Kapodistrian University of Athens (NKUA), General Hospital of Athens "Laiko"

**Research Team:**

- Christos Michailidis, Director, National Health System (NHS), General State Hospital
- Dimitrios Basoulis, Consultant B, NHS, Laiko General Hospital
- Vasileios Papastamopoulos, Director, NHS, Evangelismos Hospital
- Maria Pirounaki, Director, NHS, Hippocrates Hospital
- Garyfallia Poulakou, General Hospital for Chest Diseases "Sotiria"
- Gianna Renzio, Consultant B, NHS, General Hospital "Alexandra"
- Maria Chini, Director, NHS, Red Cross Hospital
- Georgios Chrysos, Director, NHS, Tzaneio Hospital

(Confirmation pending from two additional hospitals)

**Current Situation**

- Every patient with a positive PCR test for SARS-CoV-2 is considered a COVID-19 case and is hospitalized in a COVID Clinic.
- In Greece, every death occurring in a patient with a positive PCR test is recorded as a COVID-19 death, following WHO definitions, regardless of whether the SARS-CoV-2 infection contributed to the death.
- With the emergence of the Omicron variant:
  - There has been a vast spread of infection.
  - Many patients admitted for other reasons are found positive for SARS-CoV-2 during routine screening and are transferred to a COVID Clinic for isolation, regardless of whether they exhibit symptoms related to COVID-19.
  - In cases where these patients die, they are recorded as COVID-19 deaths, even if they had no symptoms related to COVID-19 and never received specific treatment for it.
  - The mortality attributed to COVID-19 (attributed mortality) is unknown, as surveillance focuses on crude mortality among individuals with a positive SARS-CoV-2 PCR test.

**Existing Knowledge**

- The Danish Public Health Institute (Statens Serum Institute) conducted a retrospective analysis of death certificates from February-March 2022, examining whether deaths were due to COVID-19 or merely associated with it. The findings:
  - **45% of deaths were due to COVID-19.**
- A retrospective study conducted at Laiko General Hospital analyzed all deaths in regular wards (excluding ICUs) during the Omicron wave (1/1/22 – 15/4/22). Among 1,026 admissions, 126 deaths were recorded:
  - 41 deaths were due to COVID-19.
  - 25 deaths were from other causes but involved COVID-19.
  - 60 deaths (47.6%) were with COVID-19.

(Source: Samara S, et al., Study of Causes of Death in COVID-19 Regular Wards during the Omicron Wave, 21st Panhellenic Infectious Diseases Conference, Athens, May 5-8, 2022, oral presentation.)

**Study Objective**

To retrospectively investigate the causes of death among patients who died in regular COVID-19 wards in selected hospitals during the Omicron wave.

**Key Question:** Did patients die **WITH COVID-19** or **DUE TO COVID-19**?

**Methods**

- Retrospective study of deaths occurring in COVID-19 Wards (excluding ICUs) from **January 1, 2022, to August 31, 2022**.
- Data sources for each deceased patient:
  - Death certificate
  - Medical and nursing records
  - Treating physician’s opinion
- Each death will be classified as:
  - **(a)** Directly due to COVID-19
  - **(b)** Due to another cause, but COVID-19 contributed to death
  - **(c)** Due to another disease, where SARS-CoV-2 infection played no role (**death WITH COVID-19**)

A death will be classified as **"WITH COVID-19"** if all the following conditions are met:

1. The patient was admitted for a reason unrelated to SARS-CoV-2 infection.
2. The patient tested positive for SARS-CoV-2 during routine screening.
3. The patient had no symptoms, signs, or laboratory findings compatible with COVID-19.
4. The patient did not receive COVID-19-specific treatment.
5. The death certificate lists another primary cause of death.
6. The treating physician states that the patient died from another cause.

**Interpretation of Findings**

The working hypothesis is that a significant proportion of mortality observed in patients with a positive SARS-CoV-2 PCR test during the Omicron wave is **not attributed to COVID-19** but rather to underlying conditions.

**Case Reporting Form (CRF)**

For all deaths recorded in the hospital’s regular COVID wards from **January 1, 2022, to July 31, 2022**, the following data will be collected:

**Patient Information**

- **Hospital:**
- **Patient initials:**
- **Age:**
- **Gender:**
- **Underlying Conditions:**
  - Chronic heart disease
  - Chronic respiratory disease
  - Malignancy
  - Hematologic malignancy (specify)
  - Autoimmune disease
  - Diabetes mellitus
  - Chronic kidney disease
  - Chronic liver disease
  - Chronic neurological disease/dementia
  - Fracture
  - Immunosuppression
  - Transplant recipient
  - Other (specify)

**Medical History**

- **COVID-19 Vaccination Status:** (Doses received, date of last dose)
- **Previous SARS-CoV-2 infection:** **Yes (date)** / **No**

**Hospitalization Details**

- **Admission date:**
- **Reason for admission:**
- **Initial admitting department:**
- **Date of SARS-CoV-2 diagnosis:**

**Clinical Information**

- Symptoms, signs, or lab findings consistent with COVID-19 at the time of diagnosis:
  - **No** / **Yes** (list key symptoms, e.g., hypoxia, fever, lung infiltrates, lymphopenia)
- **COVID-19-specific treatment received:**
  - **No** / **Yes**
  - Oxygen therapy: **Nasal cannula / Mask**
  - Remdesivir: **Prophylactic (3 days) / Therapeutic (5 days)**
  - Dexamethasone
  - Immunomodulators (Anakinra, Tocilizumab, Baricitinib)
  - Other antiviral medications (Nirmatrelvir/Ritonavir, Molnupiravir)

**Death Details**

- **Date of death:**
- **Cause of death (as per death certificate):**
  - **Primary cause of death:**
  - **Contributing conditions:**
- **Treating physician’s assessment:**
  - **Patient died DUE TO COVID-19**
  - **Patient died WITH COVID-19**

**Final Classification**

Based on the above criteria, each death will be classified as:

- **(a)** Due to COVID-19
- **(b)** Due to another cause but with COVID-19 as a contributing factor
- **(c)** Due to another cause, with no role of SARS-CoV-2 (**death WITH COVID-19**)

This protocol aims to accurately distinguish between deaths caused by COVID-19 and deaths where SARS-CoV-2 was an incidental finding, improving mortality surveillance and public health response strategies.
